# Effects of fermented feed of *Pennisetum giganteum* on growth performance, oxidative stress, immunity and gastrointestinal microflora of Boer goats under thermal stress

**DOI:** 10.3389/fmicb.2022.1030262

**Published:** 2023-01-12

**Authors:** Yuyang Qiu, Hui Zhao, Xiaoyu He, Furong Zhu, Fengli Zhang, Bin Liu, Qinghua Liu

**Affiliations:** ^1^National Engineering Research Center of JUNCAO Technology, Fujian Agriculture and Forestry University, Fuzhou, Fujian, China; ^2^College of Food Sciences, Fujian Agriculture and Forestry University, Fuzhou, Fujian, China; ^3^College of Animal Sciences (College of Bee Science), Fujian Agriculture and Forestry University, Fuzhou, Fujian, China

**Keywords:** thermal stress, fermented feed of *Pennisetum giganteum*, oxidative stress, immune response, gastrointestinal microflora

## Abstract

**Introduction:**

This study was conducted to evaluate the effects of fermented feed of *Pennisetum giganteum* (*P. giganteum*) on growth performance, oxidative stress, immunity and gastrointestinal microflora of Boer goats under thermal stress.

**Methods:**

The study was conducted during 45 days using twenty 2 months Boer goats. The goats were randomly allocated into two groups: NPG (*n* = 10; normal *P. giganteum*) and FPG (*n* = 10; fermented feed of *P. giganteum*), and the ratio of concentrates to roughage was 3:2. Both groups of animals were kept in sheds and exposed to summer thermal stress from 10:00 h to 18:00 h (temperature and humidity index, THI > 78). At the end of the study, the animals were slaughtered and assessed for various characteristics.

**Results:**

The findings from the study revealed that FPG-feeding significantly increased (*p* < 0.05) average daily gain (ADG, 48.18 g) and carcass weight (4.38 kg), while decreased (*p* < 0.01) average daily feed intake (ADFI, 0.74 kg/d; *p* < 0.01) and the feed:gain (F/G, 15.36) ratio. The CAT, GSH-Px activities and GSH in serum, liver and spleen, and the levels of IgA, IgG, IgM, IL-2, IL-4 and IL-1β in serum of FPG-fed goats were significantly higher (*p* < 0.05) than those of NPG-feeding goats. Further, we found that FPG feed is rich in nutrients with *Lactobacillus* (65.83%) and *Weissella* (17.80%). Results for gastrointestinal microbiota composition showed that FPG-feeding significantly enhanced the abundance of *Lactobacillus* and *unidentified Clostridiales*, and reduced *Anaerovibrio* and *Methanobrevibacter*. Meanwhile, Spearman’s correlation analysis showed that these microbiotas were closely related to the improvement of oxidative stress and immune indexes of goats.

**Discussion:**

These results demonstrated that FPG-feeding not only reduces oxidative stress and improves ROS clearance to enhance antioxidant defense system, but also improves gastrointestinal microbiota to enhance immune function by overcoming the adverse effects of heat stress, and further improve growth performance of goats.

## Highlights

- It is first time to study the feeding of Boer goats with the fermented feed of *P. giganteum* (FPG) under thermal stress in summer.
- Fermented feed of *P. giganteum* (FPG) in Boer goats’ diets improved growth performance, alleviated oxidative stress, enhanced immune defense function, and gastrointestinal microflora.
- The growth performance and health of small ruminants are closely related to the stability of gastrointestinal microflora.

## Introduction

Small ruminants, especially goat kids, play a significant part in the livestock industry (Abioja et al., 2023). Goat kids have the characteristics of fast growth, strong adaptability and plasticity. Improper daily feeding or environmental climate factors will not only affect the growth and growth performance of lambs, but also cause disease to increase mortality of lambs. Among all climatic variable, thermal stress appears to be the most critical factor that adversely affects the growth and reproductive capacity of small ruminants (Aboul Naga et al., 2021a). High humidity and high temperature not only reduce feed intake to change digestive function, but also affect the animal’s immune system and physiological functions (Rashamol et al., 2018). Therefore, in addition to improving the basic environment of the goat house, improving the immune and antioxidant function of young animals, reducing mortality and improving production performance by regulating feed nutrition has become a research hotspot in animal husbandry.

The gastrointestinal tract of ruminants is a very complex ecosystem, in which the development and microflora composition have a close effect on the establishment of digestion and absorption function, growth performance and health statue of the body (Zeineldin et al., 2018; Diao et al., 2019; Li et al., 2021). Bickhart and Weimer (2018) found that rumen microorganisms effectively convert carbohydrates into VFAs (such as acetate, propionate, and butyrate), and these acids are considered the most important because they reverse regulate the composition of rumen microorganisms, further affecting the body’s immunity. Gut microbiota promoted immunity and health by breaking down complex subunits (such as carbohydrates and proteins) and regulating metabolic mechanisms in cells (Armstrong et al., 2019). Especially, diet (feed composition) and environmental changes are the key factors affecting gastrointestinal microbiota, and can directly determine its own metabolism and immunity (Guo et al., 2022).

*Pennisetum giganteum* (*P. giganteum*), a perennial erect tufted plant of tall Gramineae, has thick and large leaves and rich in sugar, lignin, crude protein and crude fiber, so it has the reputation of good palatability and high nutrition (Peng et al., 2014). Moreover, due to its strong stress resistance, high temperature resistance, drought resistance, salt and alkali resistance, *P. giganteum* is easy to plant with high yield, which can effectively improve the shortage of forage resources and further reduce the feed grain (Hayat et al., 2020; Li et al., 2020). It has been shown that *P. giganteum* can replace corn silage, *Sorghum sudanense* and other conventional forages as livestock feed (Li et al., 2019). Therefore, *P. giganteum* is the best choice for high-quality feed for animal husbandry. The high lignocellulosic content of *P. giganteum* may be detrimental to the digestion and utilization of carbohydrates by ruminants, while feed fermented by microorganisms such as probiotics has become the most important method to improve feed quality (Zheng et al., 2020; Khan et al., 2021). Studies have shown that metabolites of microbial fermented feed have advantages of improving feed nutrition and digestion absorption rate, as well as regulating the microecological balance of rumen and intestinal tract of ruminants and improve body immunity (Metzler et al., 2005). For example, 50% *P. giganteum* silage for dairy cows, and 8 ~ 12% *P. giganteum* additive for colorful pheasant exhibit positive effects on their growth performance, digestibility (Li et al., 2020). Therefore, combining probiotics and *P. giganteum* to produce a new type of fermented feed might have higher nutritional value and development potential. Whether FPG improves immunity by regulating gastrointestinal microecology and mechanism remains to be determined.

The Boer goat breed is an important meat breed in South Africa which is well known for their good quality meat and rough-fed resistance as well as strong adaptability (Chong et al., 2019). However, little information on the effects of thermal stress on the immune function of this particular breed was available. Therefore, our study aimed to explore the effects of feeding FPG on growth performance and immunity of Boer goats under thermal stress, and to assess the possible underlying mechanisms *via* targeting on oxidative stress and inflammatory factors as well as gastrointestinal microflora, and to further analyze the relationship of gastrointestinal microflora and immune function.

## Materials and methods

### Ethics statement

The animal care, handling and sampling procedures of goats were approved by Animal Ethics Committee of the National Engineering Research Center of JUNCAO Technology and conformed to the declared plan (Ethical approval code: HC-2021-021).

### Location

**Figure 1:**
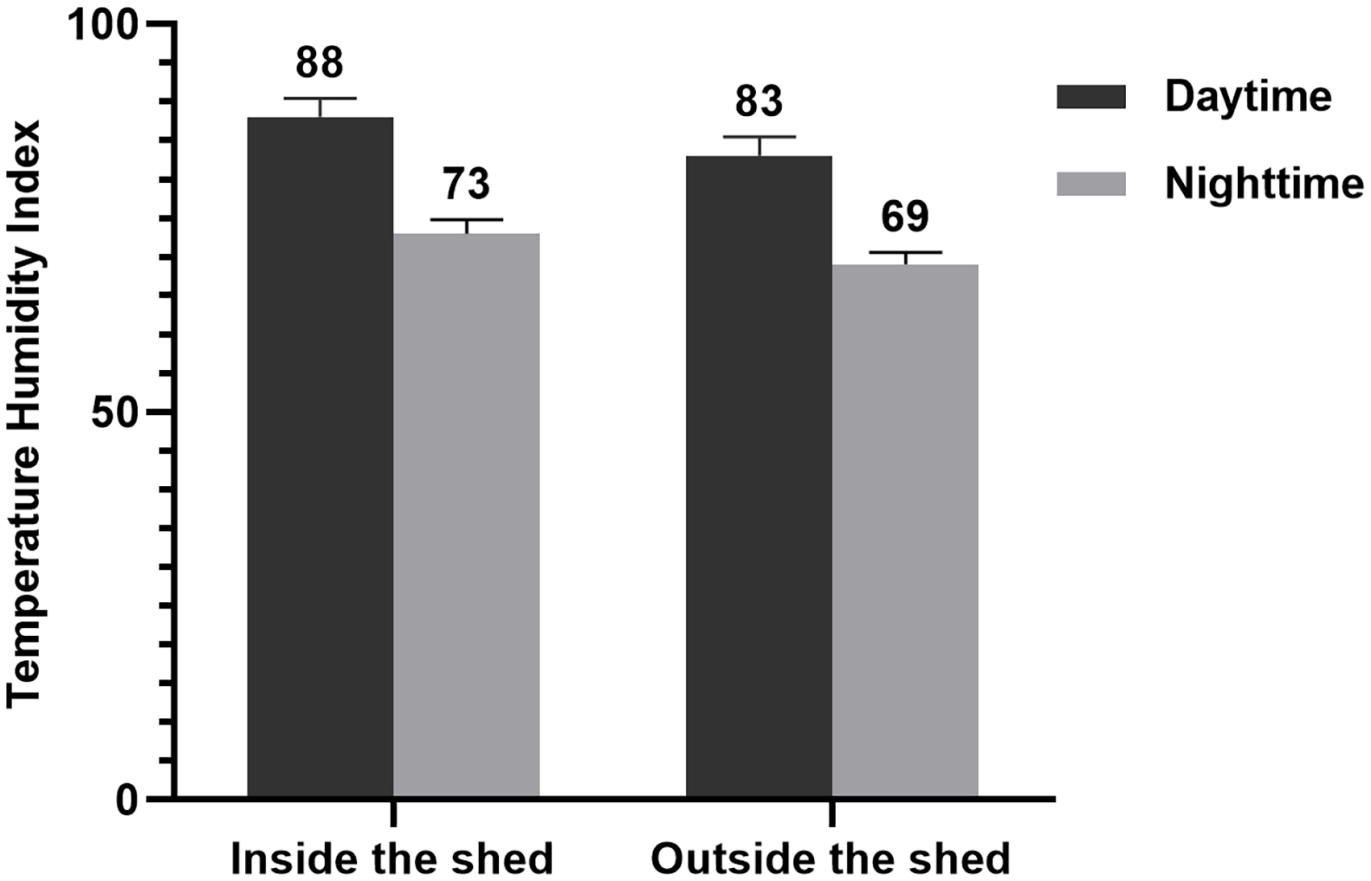
Temperature and humidity index (THI) both inside and outside the shed during the daytime and nighttime. During the daytime, the temperature exceeds 30°C for 8 h (10:00–18:00). The THI values were calculated according to Ingraham et al.’s (1974) formula. Accordingly, the formula used was THI (%) = (1.8 × temperature + 32) – (0.55–0.55 × relative humidity) × (1.8 × temperature – 26). The THI during daytime were significantly higher than at nighttime.

The study was carried out from August to October at the Breeding Farm which is located in Baizhong Town, Minqing County, Fujian Province (11°45′00″N and 39°36′36″E, 78 m above sea level). Ambient temperature and relative humidity (RH) inside and outside the sheds were recorded daily (daytime and nighttime) throughout the entire period of experiment. The temperature and humidity index (THI) values were calculated according to Ingraham et al.’s (1974) formula, and the results were depicted in Figure 1. The THI values (75 ~ 78) are indicated stressful, while those above 78 indicated great distress (Michels, 1974). The THI values obtained in this study show that Boer goats were considered to be under great (THI > 78) stress condition over 8 h during daytime.

### Preparation of fermented feed of *Pennisetum giganteum*

Culture-fermented of *P. giganteum* (Qiu et al., 2022) was formulated in the Food Biotechnology Laboratory, Fujian Agriculture and Forestry University, Fuzhou, China. *P. giganteum* (height below 2.5 m, 2-month growth cycle) were obtained from the plantation base of the National Engineering Research Center of JUNCAO Technology. Fermented film was purchased from Shanghai Bright Holstan Co., Ltd. The fermentation process of fermented feed of *P. giganteum* is as follows. Briefly, the smashed *P. giganteum* (2–3 cm), while was mixed with corn flour and wheat bran (8:1:1, a total of 3 tons), was sprayed with 2.64% culture-fermented (Qiu et al., 2022) to control the water content of the whole system at roughly 60%. After being evenly mixed, the mixture was transferred to the fermentation tank and compacted to remove air. Finally, the mixture was sealed with fermentation film and conducted at room temperature (30 ~ 40°C) for 9 days.

The contents of various nutrients in the normal and fermented feed samples of *P. giganteum* were determined before feeding trial. Nutrients mainly include dry matter, roughage protein, ether extract, roughage fiber and Ash, which the operation method was determined according to Dean et al. (2005). Detailed nutritional measurements are provided in the supplementary material. The composition is presented in Table 1.

**Table 1:** Analysis of the nutritional components of the roughage in the experimental period^1^.

Nutrition components	NPG (Control)	FPG (Treatment)	SEM^2^	*p*-Value
pH	5.89	4.07	0.08	<0.01
Dry matter %	32.52	31.78	0.03	<0.01
Crude protein %	10.03	13.90	0.14	<0.01
Ether extract %	1.77	2.13	0.06	<0.01
Crude fiber %	31.87	29.87	0.06	<0.01
Ash %	5.85	6.47	0.02	<0.01

1 Each value represents the mean of 3 replicate values.

2 SEM = standard error of the mean.

### Animals, diet and management

Twenty Boer goats (BW, 9.14  ± 0.80 kg) of 2 months of age were used for this experiment. Animals were vaccinated and dewormed for external parasites before the start of this experiment. After 2 weeks of adaptation, the goats were randomly divided into the following two groups (*n* = 10) for 45 days, which were kept in a well-ventilated shed (3 m × 4 m) under proper sanitary conditions. All animals were fed *forage mix containing concentrate (60%) and roughage (40%) ad libitum*, which coarse ratio was based on Wang et al. (2021). The concentrate was purchased from Fujian Yourong Animal Husbandry Development Co., LTD. Due to the patent of the feed formula, the proportion of each component was not disclosed, while its nutritional composition is shown in Table 2. All nutrients met the National Research Council (2007) recommendations. Two groups of goats were fed two different roughages: (1) NPG group: goats were fed with base material of *P. giganteum* as normal group, base material of *P. giganteum was composed of P. giganteum* and corn flour and wheat bran (8:1:1); (2) FPG group: goats were fed with fermented feed of *P. giganteum*. Feed was offered at 09:00 and 18:00 every day, water and salt-mineral lick were offered for *ad libitum* access to animals during the experiment. Feed amount was 4 ~ 5% of the body weight of goat and adjusted according to the feed intake of the previous day to ensure that there was unfinished feed in the feeding tank.

**Table 2:** Analysis of nutritional components of the concentrate.

Nutrition components (%)	Concentrate
Total digestible nutrients	69.38
Dry matter	90.1
Crude protein	16.58
Soluble protein	4.51
Ether extract	4.41
Neutral detergent fiber	24.78
Acid detergent fiber	11.89
Ash	7.21
Starch	20.27

Ingredients of main raw materials: Corn, soybean meal, corn germ meal, soybean peel, rumen fat powder, selenium yeast, stone powder, calcium hydrogen phosphate, trace elements (copper, iron, zinc, manganese, selenium, iodine, and cobalt), multivitamins (A, D_3_, E, B group), ethoxyquinoline, sodium propionate, etc.

### Sample collection and analysis

At the beginning of the experiment, samples of fresh *P. giganteum* (PG), base material of *P. giganteum* (NPG) and fermented feed of *P. giganteum* (FPG) were collected, frozen with liquid nitrogen and stored at −80°C.

During the experiment, the feed and residual feed amount of the two groups were accurately recorded every day. The animals were weighed on an empty stomach at 8:00 am on the 2nd day of the experiment every 15 days, the aim was to monitor their initial and final body weight (IBW and FBW) to calculate net weight (NW), average daily gain (ADG), average daily feed intake (ADFI) and feed to gain ratio (F/G).

At the end of the experimental period, the Boer goats were fasted for 12 h. Blood samples were collected into 1.5 ml sterilized tubes by jugular puncture using 10 ml disposable needle tube and placed at room temperature for 2 h. The serum was separated from the blood by centrifugation at 3000 rpm for 10 min and stored at −80°C.

The carcass was obtained from each goat after postmortem, which was killed by bloodletting through jugular vein. Then, the carcass weight, the eye muscle area and backfat thickness of the carcass were evaluated. In addition, all parts of gastrointestinal tract (compound stomach, small intestine, and large intestine), viscera and kidney fat were separated and weighed successively. The cecal contents and rumen fluid samples filtered by four layers of gauze were collected in 5 and 15 ml sterile tubes, respectively, rapidly frozen in liquid nitrogen and stored at −80°C. At the same time, part of the rumen and intestine (duodenum, caecum, and jejunum) were taken and fixed with 10% formalin. Finally, parts of liver tissue and spleen tissue from the same group were removed, cleaned with phosphate buffered saline (PBS), rapidly frozen with liquid nitrogen and stored at −80°C.

### Histopathological analysis

Rumen and intestinal samples were clipped, dehydrated, paraffin-embedded, sectioned, stained with hematoxylin and eosin, sealed and observed under a positive white light microscope (Eclipse Ci-L, Nikon, Tokyo, Japan) at 20 × or 40 × magnification.

### Biochemical assays of the serum, liver and spleen samples

#### Oxidative stress in dices in the serum, liver and spleen

The liver or spleen was mixed with normal saline at a ratio of 1:9 (*w*/*v*) to prepare homogenates using Ultra-turrax in an ice bath. The supernatant was collected after centrifugation at 1000 × *g* for 15 min at 4°C. Then, biochemical assays of the serum or liver and spleen samples were immediately measured by the kit (Nanjing Jiancheng Bioengineering Institute, China) according to the manufacturer’s instructions, including hydrogen peroxide (H_2_O_2_), malondialdehyde (MDA), lipid peroxidation (LPO), catalase (CAT), total antioxidant capacity (T-AOC), total superoxide dismutase (T-SOD), glutathione reductase (GR), glutathione peroxidase (GSH-PX), reduced glutathione (GSH).

#### Selected immunoglobulins (Ig) and interleukin in the serum

The levels of IgA, IgM, IgG, IL-2, IL-4, IL-6, and IL-1β in serum were detected by ELISA methods according to the manufacturer’s instructions (Nanjing Jiancheng Institute of Biological Engineering, China; Liu et al., 2020).

### High-throughput sequencing analysis of feed, rumen and intestinal microbiota

Total genome DNA from feed, rumen fluid and cecal contents samples were extracted using CTAB/SDS method (Cruz-Ramos et al., 2019). DNA concentration and purity were monitored on a NanoDrop 2000 UV–vis spectrophotometer (Thermo Fisher, Wilmington, MA, United States), they were checked by 1% agarose gel and diluted to 1 μg/μL with sterile water.

The V3 + V4 region of the bacterial 16S rRNA gene was amplified using 515F (5′-GTG CCA GCM GCC GCG GTA A-3′) and 806R (5′-GGA CTA CHV GGG TWT CTA AT-3′) of feed samples, 341F (5′-CCT AYG GGR BGC ASC AG −3′) and 806R of rumen fluid and cecal contents samples (Ge et al., 2020). All the libraries were constructed by TruSeq^®^ DNA PCR-Free Sample Preparation Kit (Illumina, United States), and were qualified by Qubit quantification. And then the sequencing was conducted by the Illumina NovaSeq platform. The raw tags were processed by QIIME (V1.9.1) and UCHIME algorithm (Edgar et al., 2011). The effective tags were clustered by Uparse software (Uparse v7.0.1001; Edgar, 2013) with 97% similarity cutoff to obtain OTUs (Operational Taxonomic Units). The Spearman correlation heatmap between rumen and intestinal microflora and immune and oxidative stress indicators at the genetic level was based on the RStudio software. In addition, the correlation network was visualized by Cytoscape 3.6.1 (Huang et al., 2019).

### Statistical analysis

Statistical analysis of all data was applied to investigate correlation by unpaired two-tailed Students’ *t*-test using SPSS 13.0 and GraphPad Prism 8 software. All the results are presented as the mean. The reliability of experimental data was expressed by Standard Error of Mean (SEM). The significance level of the test results was set at *p* < 0.05 and *p* < 0.01, which were considered as statistically significant and extremely significant, respectively.

## Results

### Growth performance

Both groups of Boer goats showed normal behavior throughout the 45-day experiment. As can be seen from Table 3, there was no difference in initial body weight between the two groups of Boer goats (*p* = 0.44). After the experiment, compared with NPG group, FBW of FPG group was increased by 11.44% (*p* = 0.05), NW and ADG of FPG group were extremely significantly increased by 73.6 and 82.7% (*p* < 0.01), respectively. ADFI and F/G ratio were extremely significantly decreased (*p* < 0.01).

**Table 3:** Effect of fermented feed of *Pennisetum giganteum* on growth performance of Boer goats in thermal stress^1^.

Item^2^	NPG	FPG	SEM^3^	*p*-Value
IBW, kg	9.05	9.23	0.78	0.44
FBW, kg	10.23	11.40	1.18	0.05
NW, kg	1.25	2.17	0.60	<0.01
ADG, g	26.37	48.18	13.00	<0.01
ADFI, kg/d	1.38	0.74	0.94	<0.01
F/G ratio	52.33	15.36	21.71	<0.01

1 Each value represents the mean of 3 replicate values.

2 IBW = initial body weight; FBW = final body weight; NW = net weight; ADG = average daily gain; ADFI = average daily feed intake; F/G ratio = feed to gain ratio.

3 SEM = standard error of the mean.

### Slaughter performance

The slaughter performance (Table 4) was shown that the live weight before slaughter, carcass weight and eye muscle area of FPG group were significantly higher than those of NPG group (*p* < 0.05), which were increased by 20.5, 25.86, and 94.94%, respectively. Carcass weight ratio, back fat thickness and viscera weight of FPG group were higher than those of NPG group, but there was no significant difference between the two groups (*p* > 0.05). Performance indexes of gastrointestinal tract were shown in Table 5. Small intestine weight of FPG group was extremely significantly higher than that of NPG group (*p* < 0.01), and omasum weight, abomasum weight and large intestine weight of FPG group were significantly higher than that of NPG group (*p* < 0.05). There were no significant differences between the two groups in other indexes of gastrointestinal tract detection, such as rumen wall thickness, papilla height, rumen weight and reticulum weight (*p* > 0.05).

**Table 4:** Effect of fermented feed of *Pennisetum giganteum* on slaughter performance of Boer goats in thermal stress^1^.

Item	NPG	FPG	SEM^2^	*p*-Value
Live weight before slaughter, kg	9.17	11.05	0.47	0.01
Carcass weight, kg	3.48	4.38	0.33	0.04
Carcass weight ratio, %	37.99	39.59	1.57	0.30
Backfat thickness, mm	3.17	3.56	0.45	0.47
Eye muscle area, cm^2^	10.27	20.02	3.53	0.03
Heart weight, kg	0.05	0.06	0.01	0.68
Liver weight, kg	0.17	0.19	0.01	0.07
Spleen weight, kg	0.01	0.01	0.01	0.37
Lung weight, kg	0.11	0.12	0.02	0.15
Kidney weight, kg	0.05	0.04	0.01	0.53
Perirenal fat weight, g	11.63	11.38	1.65	0.86

1 Each value represents the mean of three replicate values.

2 SEM = standard error of the mean.

**Table 5:** Effect of fermented feed of *Pennisetum* giganteum on gastrointestinal tract of Boer goats in thermal stress^1^.

Item	NPG	FPG	SEM^2^	*p*-Value
Rumen wall thickness, mm	1.29	1.18	0.41	0.76
Rumen papilla height, mm	3.18	3.22	0.31	0.89
Rumen weight, kg	0.20	0.22	0.02	0.18
Reticulum weight, kg	0.04	0.05	0.01	0.37
Omasum weight, kg	0.03	0.05	0.01	0.03
Abomasum weight, kg	0.07	0.09	0.01	0.05
Small intestine weight, kg	0.24	0.30	0.02	<0.01
Large intestine weight, kg	0.13	0.17	0.02	0.05

1 Each value represents the mean of 3 replicate values.

2 SEM = standard error of the mean.

### Histologic section analysis

**Figure 2:**
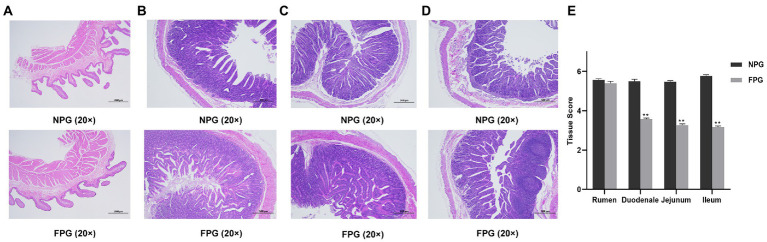
Histologic section analysis of gastrointestinal tract of Boer goats was performed at 20 × and 40 × low magnification. **(A)** the rumen tissue (20× magnification); **(B)** the duodenal tissue (40× magnification); **(C)** the jejunum tissue (40× magnification); **(D)** the ileum tissue (40× magnification); **(E)** the tissue score. Data are expressed as mean ± SD (*n* = 3). ^*^p < 0.05 and ^**^p < 0.01 compared with the NPG group.

The histological morphology of rumen sections is referred to in Figure 2A. There were no significant differences in rumen villus morphology, gastric wall thickness and lamina propria status between the two groups. Compared with NPG group, the villi distribution and growth density of duodenum, jejunum and ileum of Boer goats in FPG group were more dense and complete, and the glands were arranged neatly and distributed more regularly (Figures 2B–D). In NPG group, the duodenum and jejunum villi were slightly incomplete, and the muscle layer was thickened. The ileum villi were broken and autolysis, and the muscle layer was obviously thickened. As shown in Figure 2E, compared with the NPG group, duodenum, jejunum and ileum tissue score of FPG were significantly reduced (*p* < 0.01), only rumen tissue score has no significant difference (*p* > 0.05).

### Oxidative stress status in the serum, liver and spleen of Boer goats

The oxidant indices in Table 6 show that the contents of H_2_O_2_, MDA and LPO in serum of FPG group were decreased by 45.74% (*p* < 0.05), 4.28% (*p* > 0.05) and 14.81% (*p* < 0.05) as compared to the control group (NPG). The above three indicators in liver of FPG group were significantly decreased by 54.97, 58.97, and 59.09% (*p* < 0.05), respectively. In spleen, the content of H_2_O_2_ in FPG group was significantly decreased (*p* < 0.05), while MDA and LPO contents had no significant differences. As shown in Table 7, compared with the NPG group, the activities of CAT, GR, T-AOC (*p* < 0.05) and GSH, GSH-Px (*p* < 0.01) in serum of FPG group were significantly increased, while there was no significant difference in T-SOD. In liver and spleen, the activities of CAT, GSH and GSH-Px in FPG group were significantly increased (*p* < 0.05), while there was no significant difference in T-SOD. Interestingly, there was no significant difference between liver GR and spleen T-AOC activities, while spleen GR and liver T-AOC activities increased significantly (*p* < 0.05).

**Table 6:** Effect of fermented feed of *P. giganteum* on oxidant status in the serum, liver and spleen of Boer goats in thermal stress^1^.

Item^2^	NPG	FPG	SEM^3^	*p*-Value
Serum
H_2_O_2_, mmol/L	20.18	10.95	2.80	0.02
MDA, nmol/ml	5.37	5.14	0.17	0.23
LPO, mol/L	0.81	0.69	0.05	0.05
Liver
H_2_O_2_, mmol/gprot	17.79	8.01	3.42	0.05
MDA, nmol/mgprot	0.39	0.16	0.07	0.02
LPO, mmol/gprot	0.44	0.18	0.07	0.01
Spleen
H_2_O_2_, mmol/gprot	30.18	18.49	2.72	0.01
MDA, nmol/mgprot	1.28	0.78	0.24	0.08
LPO, mmol/gprot	0.44	0.60	0.17	0.33

1 Each value represents the mean of 3 replicate values.

2 H_2_O_2_ = hydrogen peroxide; MDA = malonaldehyde; LPO = lipid peroxidation.

3 SEM = standard error of the mean.

**Table 7:** Effect of fermented feed of *Pennisetum giganteum* on antioxidant status in the serum, liver and spleen of Boer goats in thermal stress^1^.

Item^2^	NPG	FPG	SEM^3^	*p*-Value
Serum
CAT, U/mL	0.41	2.40	0.69	0.03
GSH, U/mL	1.19	8.53	1.04	<0.01
T-SOD, U/mL	3.15	4.48	1.24	0.34
GSH-Px, U/mL	25.30	121.34	3.42	<0.01
GR, U/L	1.61	12.86	2.90	0.03
T-AOC, mM	0.38	0.43	0.01	0.02
Liver
CAT, U/mgprot	97.82	70.67	2.31	0.01
GSH, U/mgprot	2.66	4.55	0.75	0.04
T-SOD, U/μgprot	7.48	7.78	1.26	0.79
GSH-Px, U/mgprot	11.90	21.96	3.90	0.04
GR, U/gprot	5.28	6.80	1.02	0.16
T-AOC, mmol/gprot	0.03	0.04	<0.01	0.01
Spleen
CAT, U/mgprot	2.51	3.87	0.53	0.04
GSH, U/mgprot	3.26	6.45	1.27	0.04
T-SOD, U/μgprot	6.23	8.58	1.42	0.15
GSH-Px, U/mgprot	34.08	84.63	3.69	0.01
GR, U/gprot	3.77	8.22	0.73	<0.01
T-AOC, mmol/gprot	0.09	0.13	0.02	0.13

1 Each value represents the mean of 3 replicate values.

2 CAT = catalase; GSH = glutathione; T-SOD = total superoxide dismutase; GSH-Px = glutathione peroxidase; GR = glutathione reductase.

3 SEM = standard error of the mean.

### Serum immune parameters of Boer goats

At the end of 45 days of feeding, different immune indexes were observed in Table 8. The contents of IgA, IgG, IgM, IL-2, IL-4, and IL-1β in serum of FPG group were significantly higher than those of NPG group (*p* < 0.05). Furthermore, the levels of IL-6 increased and decreased without any significant difference.

**Table 8:** Effect of fermented feed of *Pennisetum giganteum* on serum immune parameters of Boer goats in thermal stress^1^.

Item^2^	NPG	FPG	SEM^3^	*p*-Value
IgA, g/L	3.06	3.63	0.15	0.01
IgG, g/L	87.27	137.95	1.51	< 0.01
IgM, g/L	1.35	3.32	0.43	0.01
IL-2, mg/L	0.33	0.42	0.03	0.02
IL-4, mg/L	0.59	0.79	0.08	0.03
IL-6, ng/L	28.70	28.38	0.59	0.55
IL-1β, ng/L	4.80	9.92	0.36	< 0.01

1 Each value represents the mean of 3 replicate values.

2 Ig = immunoglobulin; IL = interleukin.

3 SEM = standard error of the mean.

### Structure of feed and gastrointestinal microbial communities

**Figure 3:**
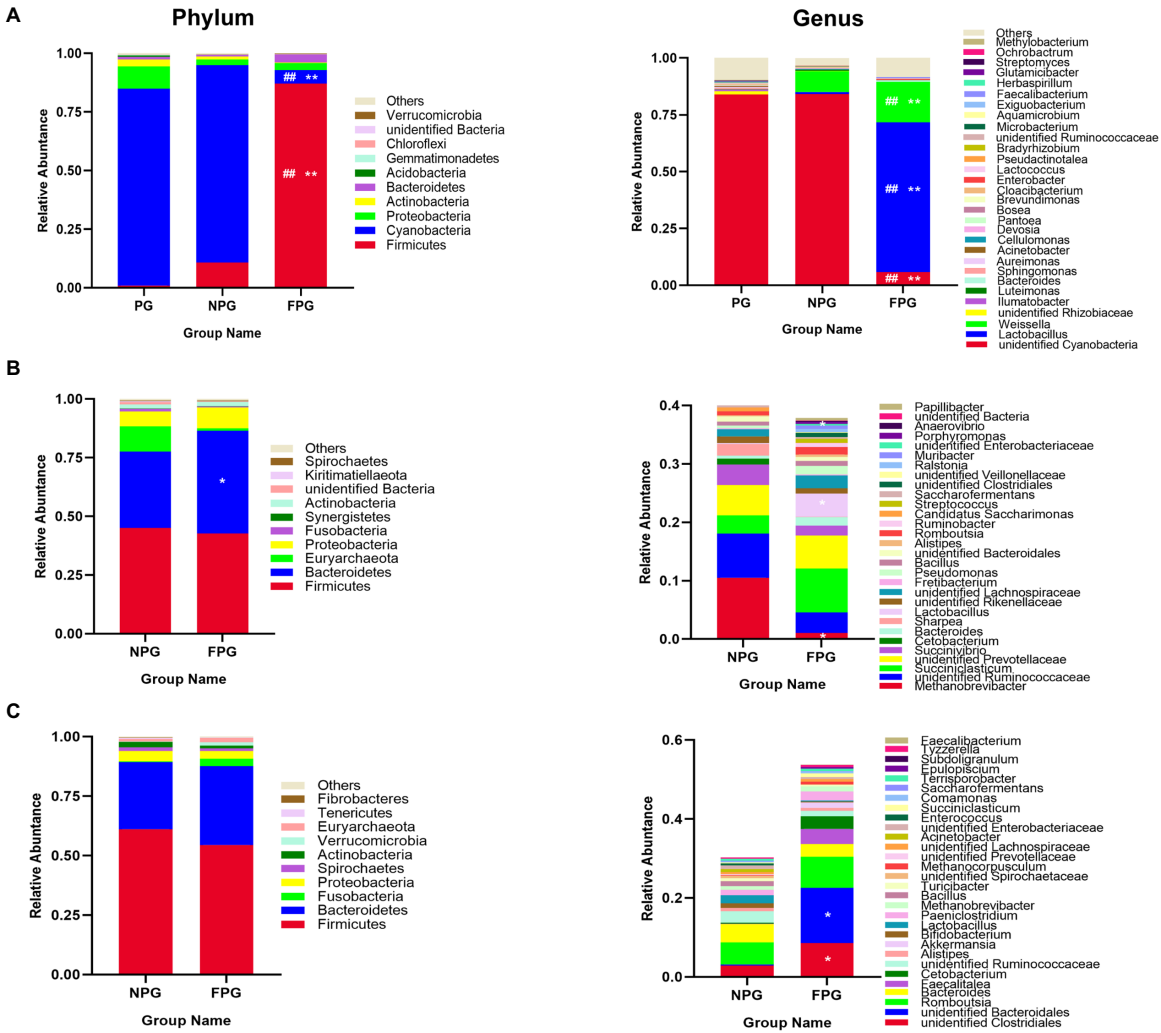
Histogram of the relative abundance of feed and gastrointestinal microbiota at the phylum (Left column) and genus (Right column) level. **(A)** Feed. **(B)** Rumen. **(C)** Caecum contents. Data are expressed as mean ± SD (*n* = 3). ^#^*p* < 0.05 and ^##^*p* < 0.01 compared with the PG group; ^*^p < 0.05 and ^**^p < 0.01 compared with the NPG group.

Relative percentages of dominant taxa at phylum and genus levels were assessed by feed, rumen and intestinal microbiota (Figure 3). The abundance of Firmicutes (86.95%), *Lactobacillus* (65.83%) and *Weissella* (17.80%) in FPG feed was significantly higher (*p* < 0.01) than that in PG feed and NPG feed (Figure 3A). Additionally, the rumen microbiota of NPG (45.04, 32.57, 10.64, and 6.41%) and FPG (42.63, 43.78, 1.08, and 8.90%) group were predominated by Firmicutes, Bacteroidetes, Euryarchaeota and Proteobacteria as the dominant microflora, other phyla (Fusobacteria, Synergistetes, Actinobacteria and Spirochaetes) were indicated with a lower abundance (Figure 3B). Interestingly, *Lactobacillus* in FPG group (3.94%) were significantly higher (*p* < 0.05), while *Anaerovibrio* and *Methanobrevibacter* was significantly lower (*p* < 0.05) than that in NPG group. Lastly, the intestinal microbiota of NPG and FPG group were composed mainly by the phyla Firmicutes (61.07, 54.44%), Bacteroidetes (28.23, 33.07%) and Proteobacteria (4.37%, 3. 27%), respectively (Figure 3C). Other phyla (Spirochaetes, Actinobacteria, Verrucomicrobia, Euryarchaeota, and Tenericutes) were all at a low abundance level. At the genus level (Figure 3C), the relative abundance of *unidentified Clostridiales*, *Romboutsia* and *Akkermansia* in FPG group (8.56, 7.89, and 1.29%) were higher than those in NPG group (2.90, 5.59, and 0.04%).

### Correlations of the gastrointestinal microbiota with immune and oxidative stress parameters

**Figure 4:**
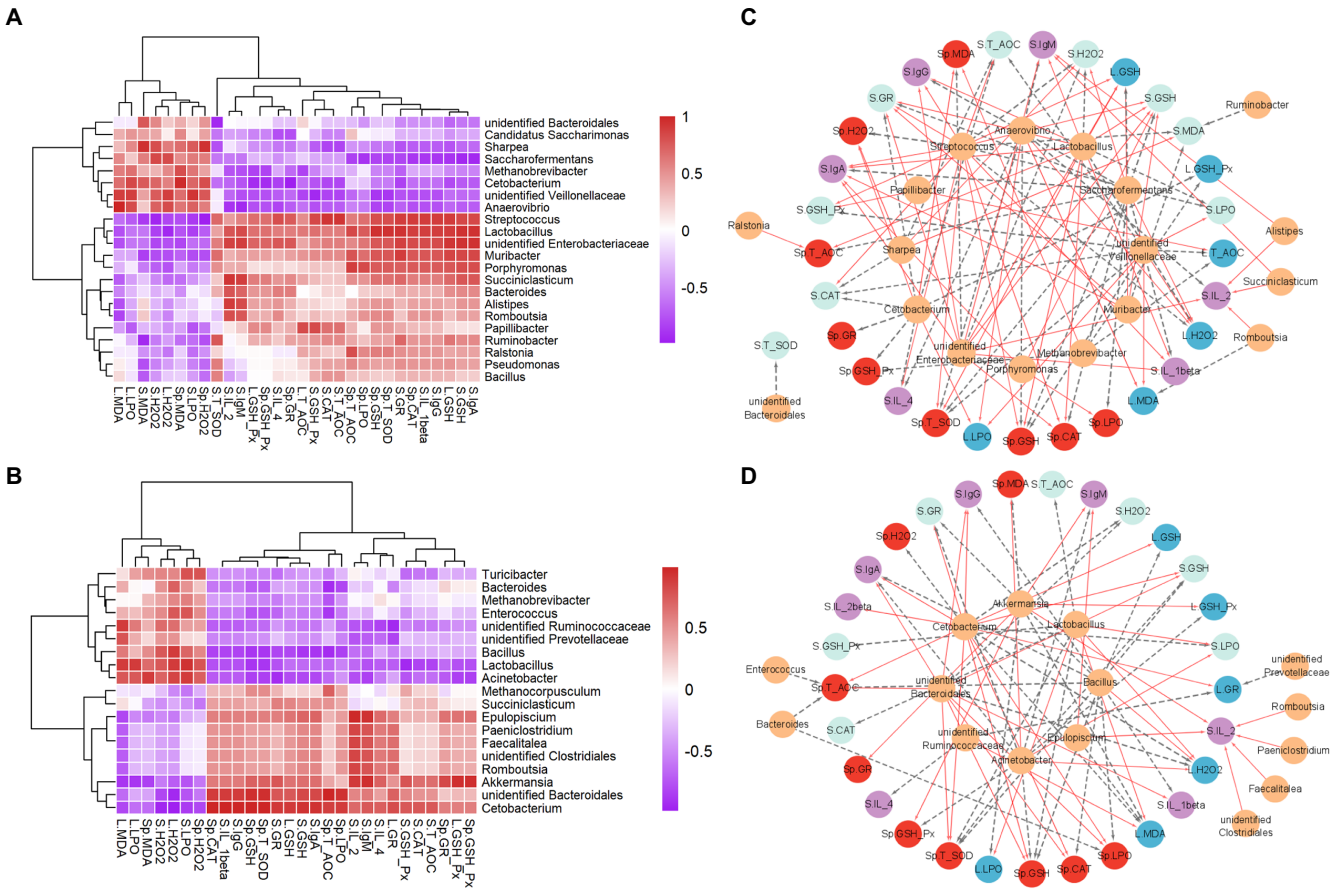
Statistical Spearman’s correlations between the rumen **(A,C)** or gut microbiota **(B,D)** and immune and oxidative stress parameters in NPG and FPG groups. **(A)** Heatmap of correlation between the rumen microbiota and immune and oxidative stress parameters. **(B)** Heatmap of correlation between the gut microbiota and immune and oxidative stress parameters. The intensity of the color represents the degree of association among them. **(C,D)** Visualization of the correlation network according to the correlation. Yellow nodes, the rumen microbial genera **(C)**, the gut microbial genera **(D)**; red nodes, the spleen parameters; blue nodes, liver parameters; green nodes, the serum oxidative stress parameters; purple nodes, serum immune parameters. Red lines, Spearman’s rank correlation coefficient > 0.8, FDR adjusted *p* < 0.05; dotted gray lines, Spearman’s rank correlation coefficient < − 0.8, FDR adjusted *p* < 0.05. The line width indicates the strength of correlation.

The correlations between the gastrointestinal microbiota and immune and oxidative stress related parameters were calculated using Spearman correlation analysis (Figures 4A,B) and visualized by network (Figures 4C,D). We found that *Methanobrevibacter* was positively correlated with spleen MDA, but negatively correlated with spleen GSH-Px and liver GSH-Px, T-AOC (Figures 4A,C). Moreover, *Anaerovibrio* was negatively correlated with serum indexes (IL-2, IL-4, and IgM) and spleen indexes (GSH, T-SOD), but positively correlated with liver (H_2_O_2_, MDA, and LPO). More interestingly, the *Lactobacillus* was positively associated with serum GSH, GR, IgA, IgG, IgM, liver GSH, spleen CAT, GSH, T-SOD, T-AOC, but negatively related to serum H_2_O_2_, LPO, and liver H_2_O_2_. In addition, serum IL-2 had a positive correlation with *Succiniclasticum*, *Alistipes*, *Romboutsia* and *unidentified Enterobacteriaceae*. Results (Figures 4B,D) showed that *unidentified Clostridiales*, *Romboutsia*, *Faecalitalea* and *Akkermansia* were positively correlated with serum IL-2. Interestingly, *Akkermansia* was positively associated with liver and spleen IgM, GSH-Px, and spleen GR, while was negatively correlated with liver MDA, LPO, spleen H_2_O_2_.

## Discussion

Under high values of ambient temperature and humidity, the heat dissipation ability of animals is reduced, resulting in increased body temperature. The THI value was proposed to detect animals under thermal stress (Jaber et al., 2019). The experiment was conducted in the summer of Fujian province, and the THI value was higher than 75 with continuous high temperature (> 30°C) for 8 h inside and outside the breeding shed during the day. Therefore, the breeding was considered to be carried out under thermal stress. In subtropical conditions, the modifications of diet composition should be used to promote a higher intake or compensate the low feed consumption (Muklada et al., 2020).

After a short fermentation period of 9-day, the pH and crude fiber content of feed (FPG) were significantly decreased (*p* < 0.01), while crude protein, crude fat content and abundant microorganisms such as *Lactobacillus* and *Weissella* were significantly increased (*p* < 0.01). Reports indicate that the roughage with high protein and low crude fiber had better palatability, which was beneficial to animal digestion and absorption, thus improving the carcass quality (Ma et al., 2020). This was consistent with the results of trial. The indicators of ADG, carcass weight and eye muscle area of goats fed FPG were significantly increased. Studies have shown that the unique acid and wine aroma of microbial fermented feed induced goats to adopt feed (Cui et al., 2019). It has been reported that *Lactobacillus* and *Weissella* can increase the abundance of short-chain fatty acids (SCFAs), and stimulate the development of the gastrointestinal tract by adjusting gastrointestinal flora structure, thereby improve inflammation (He et al., 2022). These findings also support the current results. The weight of omasum, abomasum, small intestine and large intestine of FPG group were significantly higher, and the rumen and intestinal tissue structure were also better than that of NPG group. The observed improvements in ADG and F:G could be attributed to the synergistic effect of fermentative metabolites in FPG feed with compound probiotics. Therefore, FPG feed with higher nutritional quality and shorter fermentation time were superior to NPG feed and conventional silage (Du et al., 2022). Previous studies have shown that thermal stress reduces goat growth performance (Live weight and carcass weight), which could be attributed to the severe energy expenditure of the animal to resist high heat (Abhijith et al., 2021) and both increased respiratory and cutaneous evaporative cooling mechanisms (Aboul Naga et al., 2021b). Therefore, it is normal that the growth performance of goats in this trail is slightly lower than that of previous studies (Mu et al., 2019).

Previous studies have confirmed that probiotics would regulate systemic inflammation and oxidative stress (Bohlouli et al., 2021; Zheng et al., 2021). Moreover, the oxidative stress caused by intracellular reactive oxygen species (ROS) would also activate the corresponding antioxidant defense system, which is composed of enzyme components such as CAT, GR and GSH-Px and non-enzyme components such as T-SOD, GSH and T-AOC (Landry et al., 2015). It was reported that feeding *Lactobacillus plantarum*-treated silage increased dairy goat’s serum antioxidase activity, such as T-AOC, SOD, GSH-Px and CAT (Li et al., 2022). This was consistent with the results of serum, liver and spleen fed FPG in this study. In addition, the unique acid flavor of compound bacterial feed could not only induce the increase of feed intake of lambs, but also cause stress to the body (Cui et al., 2019). SOD could transform superoxide free radicals into H_2_O_2_, so the lower SOD activity was a protective effect on the body. This can explain the reason of no difference in T-SOD. Therefore, the supplementation of FPG in the diets effectively enhanced the antioxidant defense system and improved the capability to scavenge ROS of goats.

Thermal stress has been reported to inhibit immune function of ruminants (Abioja et al., 2023). Serum immunoglobulins secreted by B cells are the main effective molecule, which moderately reflects the actual humoral immunity (Liu et al., 2020). Cytokines are secreted by activated immune cells such as Th1 cells, which can interact with each other to form a signal network and play a role in regulating the host immune level (Zeng et al., 2021). Previous studies have shown that probiotics might affect the secretion of immunoglobulin (IgA, IgM, and IgG) and cytokines (IL-2 and IL-4) by immune organs and cells, thus improving the immune function of the body (Alizadeh et al., 2022; Li et al., 2022). This is consistent with the results (FPG) of this study. IL-6 and IL-1β are mainly produced by macrophages/monocytes in fat or peripheral tissues, which mediate tissue-specific inflammation (Akash et al., 2017). Probiotics such as *Lactobacillus* and *Bacteroidetes* can induce dendritic cells and macrophages to produce TNF-α, IL-1β and IL-6 to act as pro-inflammatory factors (Dwivedi et al., 2016). The appropriate increase of these pro-inflammatory factors can stimulate the secretion of IgG, and promote the activation of B and T cells to regulate the body’s immune function (Ma et al., 2018). Thus, FPG diet decreased proinflammatory factors, increased anti-inflammatory factors and improved immune disorders in goats.

We detected *Lactobacillus*, *unidentified Clostridium*, *Anaerovibrio* and *Methanobrevibacter* key gastrointestinal microflora, by performing the relationship between oxidative stress, immunity and gastrointestinal microflora indicators through Spearman’s correlation analysis. Many studies have reported that the dominant bacteria in the gastrointestinal tract of ruminants are Firmicutes and Bacteroidetes (Cremonesi et al., 2018; Mu et al., 2019; Chen et al., 2020). Firmicutes are the main bacteria that decompose fiber and cellulose in the rumen, while Bacteroidetes are mainly responsible for carbohydrate and protein digestion and are conducive to the maturation of the gastrointestinal immune system (Li et al., 2021). Ge et al. (2020) found that the lower ratio of Firmicutes to Bacteroidetes (F/B) increase the relative abundance of certain microorganisms, so as to alleviate the lipid metabolism disorders and promote health. This is consistent with the results of this study. The F/B ratios in gastrointestinal tract in NPG group were 1.38 and 2.16, while those in FPG group were 0.97 and 1.65, respectively. Therefore, gastrointestinal microbes with a low F/B ratio of fed-FPG are more conducive to relieving oxidative stress and improving immunity. This view was verified by the fact that the bacteria at the genus level discussed in this study belong to these two phyla. *Lactobacillus*, fastidious Gram-positive bacteria, are generally considered as commensal or an indicator of healthy microbiota (Fijan, 2014). It has been demonstrated that *Lactobacillus* are capable to ferment oligo- and poly-saccharides in in rumen to produce organic acids, and regulate rumen pH (Li et al., 2021). And it can also show some antibacterial effects through producing organic acids and antimicrobial peptides competing with pathogenic bacteria for sites (Kim et al., 2020). *Unidentified Clostridiales* are responsible for the degradation of various substrates (sugars and proteins) as well as the production of volatile fatty acids (Yi et al., 2014). Studies have shown that these fatty acids have special antibacterial activity, which could inhibit intestinal pathogens and reduce inflammation to replace antibiotics (Li et al., 2022). *Anaerovibrio* is a group of lipid-degrading bacteria, which is prone to produce oxidizing substances such as malondialdehyde and 4-hydroxy-2-hexenal (Sun et al., 2022). The oxidation products would affect intestinal absorption and digestion with a strong proinflammatory effect. Additionally, as Eurycoarchaea, *Methanobrevibacter* is the main methanogenic bacteria in the rumen. The higher its abundance, the more energy it releases through methanogenesis (Tymensen et al., 2012). This is also the reason for the poor growth performance of goats in NPG group.

The above experiments indicated that the decrease of various biochemical indexes in goats under thermal stress conditions resulted in the immunosuppression of goats. FPG diet can improved the immunity of goats, as evidenced by remission of disease phenotype, the restoration of intestinal microecology, and improvement of growth performance. In terms of the correlation between gastrointestinal microbiota and immunity, FPG feed rich in beneficial bacteria, such as *Lactobacillus* and *Weissella*, accumulated in the gastrointestinal tract of goats and improved the microecology of gastrointestinal microbiota (such as elevated *Lactobacillus*, *unidentified Clostridiales*). Furthermore, FPG significantly attenuated oxidative stress of liver and spleen and improved its antioxidant capacity. Finally, FPG ameliorated cytokines of serum to enhance immune function. However, we have only been able to demonstrate that FPG diets improve immune function through regulating the gastrointestinal microbiota, and further research is needed on how the gastrointestinal microbiota works.

## Conclusion

In conclusion, this experiment highlighted the effects of thermal stress on immunocompromised Boer goat models under temperate environment. The results indicate that FPG-feeding improves the growth performance and biochemical indices of Boer goats. Besides, FPG-feeding effectively enhanced antioxidant defense system and immune function through reducing hepatolienal oxidative stress and increasing the scavenging ability of ROS, as well as ameliorating cytokines of serum and the homeostasis of gastrointestinal microbiotas. FPG will have a good application prospect in replacing antibiotics acted as a functional feed. These investigations provide new experimental evidence for the future application of FPG diets in goats.

## Data availability statement

The datasets presented in this study can be found in online repositories. The data of 16s sequence data presented in the study are deposited in the NCBI repository, accession number PRJNA922864. The original contributions of nutritional measurements presented in the study are included in the article/Supplementary material, further inquiries can be directed to the corresponding author.

## Ethics statement

The animal study was reviewed and approved by Animal Ethics Committee of the National Engineering Research Center of JUNCAO Technology. Written informed consent was obtained from the owners for the participation of their animals in this study.

## Author contributions

YQ: investigation, resources, formal analysis, writing: original draft preparation. HZ: investigation, visualization, and supervision. XH and FuZ: data curation. FeZ: validation. BL and QL: conceptualization, methodology, funding acquisition, supervision, and writing: review and editing. All authors contributed to the article and approved the submitted version.

## Funding

This work was financially supported by grants from Fujian Agriculture and Forestry University International Cooperation and Exchange Project (No. KXG15001A), Science and Technology Innovation Fund Project of Fujian Agriculture and Forestry University (KFA18055A and KFA18056A), Fungus and Grass Science and Industry Development Project (XKJC-712021030), Central Leading Local Science and Technology Development Fund of Fujian Province (2022L3085).

## Conflict of interest

The authors declare that the research was conducted in the absence of any commercial or financial relationships that could be construed as a potential conflict of interest.

## Publisher’s note

All claims expressed in this article are solely those of the authors and do not necessarily represent those of their affiliated organizations, or those of the publisher, the editors and the reviewers. Any product that may be evaluated in this article, or claim that may be made by its manufacturer, is not guaranteed or endorsed by the publisher.

## References

[ref1] Abhijith A. Sejian V. Ruban W. Krishnan G. Bagath M. Pragna P.. (2021). Summer season induced heat stress associated changes on meat sproduction and quality characteristics, myostatin and HSP70 gene expression patterns in indigenous goat. Small Ruminant Res. 203:106490. doi: 10.1016/j.smallrumres.2021.106490

[ref2] Abioja M. O. Logunleko M. O. Majekodunmi B. C. Adekunle E. O. Shittu O. O. Odeyemi A. J.. (2023). Roles of candidate genes in the adaptation of goats to heat stress: a review. Small Ruminant Res. 218:106878. doi: 10.1016/j.smallrumres.2022.106878

[ref3] Aboul Naga A. M. Abdel Khalek T. M. Osman M. Elbeltagy A. R. Abdel-Aal E. S. Abou-Ammo F. F.. (2021a). Physiological and genetic adaptation of desert sheep and goats to heat stress in the arid areas of Egypt. Small Ruminant Res. 203:106499. doi: 10.1016/j.smallrumres.2021.106499

[ref4] Aboul Naga A. M. Elshafie M. H. Khalifa H. Osman M. Abdel Khalek T. M. El Beltagi A. R.. (2021b). Tolerance capability of desert sheep and goats to exercise heat stress under hot dry conditions, and its correlation with their production performance. Small Ruminant Res. 205:106550. doi: 10.1016/j.smallrumres.2021.106550

[ref5] Akash M. H. Rehman K. Liaqat A. (2017). Tumor necrosis factor-alpha: role in development of insulin resistance and pathogenesis of type 2 diabetes mellitus. J. Cell. Biochem. 119, 105–110. doi: 10.1002/jcb.26174, PMID: 28569437

[ref6] Alizadeh M. Astill J. Alqazlan N. Shojadoost B. Taha-Abdelaziz K. Bavananthasivam J.. (2022). In ovo co-administration of vitamins (A and D) and probiotic lactobacilli modulates immune responses in broiler chickens. Poultry Sci. 101:101717. doi: 10.1016/j.psj.2022.101717, PMID: 35172231 PMC8851267

[ref7] Armstrong L. E. Casa D. J. Belval L. N. (2019). Metabolism, bioenergetics and thermal physiology: influences of the human intestinal microbiota. Nutr. Res. Rev. 32, 205–217. doi: 10.1017/S0954422419000076, PMID: 31258100

[ref8] Bickhart D. M. Weimer P. J. (2018). Symposium review: host-rumen microbe interactions may be leveraged to improve the productivity of dairy cows1. J. Dairy Sci. 101, 7680–7689. doi: 10.3168/jds.2017-13328, PMID: 29102146

[ref9] Bohlouli J. Namjoo I. Borzoo-Isfahani M. Kermani M. A. H. Zehi Z. B. Moravejolahkami A. R. (2021). Effect of probiotics on oxidative stress and inflammatory status in diabetic nephropathy: a systematic review and meta-analysis of clinical trials. Heliyon 7:e5925:e05925. doi: 10.1016/j.heliyon.2021.e05925, PMID: 33490683 PMC7808957

[ref10] Chen G. J. Zhang R. Wu J. H. Shang Y. S. Xiong X. Q. (2020). Effects of soybean lecithin supplementation on growth performance, serum metabolites, ruminal fermentation and microbial flora of beef steers. Livest. Sci. 240:104121. doi: 10.1016/j.livsci.2020.104121

[ref11] Chong Y. Liu G. Jiang X. Fu Y. Liu C. Bo D. (2019). Genetic introgression of Boer goat into indigenous goat breeds in the three gorges area of China. Small Ruminant Res. 176, 1–4. doi: 10.1016/j.smallrumres.2019.05.001

[ref12] Cremonesi P. Conte G. Severgnini M. Turri F. Monni A. Capra E.. (2018). Evaluation of the effects of different diets on microbiome diversity and fatty acid composition of rumen liquor in dairy goat. Animal 12, 1856–1866. doi: 10.1017/S1751731117003433, PMID: 29306345

[ref13] Cruz-Ramos J. Hernández-Triana L. Pea G. Gonzalez-Alvarez V. H. Ortega-Morales A. I. (2019). Comparison of two DNA extraction methods from larvae, pupae, and adults of Aedes aegypti. Heliyon 5:e2660:e02660. doi: 10.1016/j.heliyon.2019.e02660, PMID: 31692696 PMC6806409

[ref14] Cui Y. Li X. Q. Yu C. W. Li D. F. Gao M. Hu H. L.. (2019). Effects of compound bacteria culture on growth performance, immunity and antioxidant function of meat sheep. Anim. Nutr. 31, 5065–5073. doi: 10.3969/j.issn.1006?267x.2019.11.021

[ref15] Dean D. B. Adesogan A. T. Krueger N. Littell R. C. (2005). Effect of Fibrolytic enzymes on the fermentation characteristics, aerobic stability, and digestibility of Bermudagrass silage. J. Dairy Sci. 88, 994–1003. doi: 10.3168/jds.S0022-0302(05)72767-3, PMID: 15738234

[ref16] Diao Q. Zhang R. Fu T. (2019). Review of strategies to promote rumen development in calves. Animals 9:490. doi: 10.3390/ani9080490, PMID: 31357433 PMC6720602

[ref17] Du Z. Sun L. Lin Y. Chen C. Yang F. Cai Y. (2022). Use of Napier grass and rice straw hay as exogenous additive improves microbial community and fermentation quality of paper mulberry silage. Anim. Feed Sci. Tech. 285:115219. doi: 10.1016/j.anifeedsci.2022.115219

[ref18] Dwivedi M. Kumar P. Laddha N. C. Helen Kemp E. (2016). Induction of regulatory T cells: a role for probiotics and prebiotics to suppress autoimmunity. Autoimmun. Rev. 15, 379–392. doi: 10.1016/j.autrev.2016.01.002, PMID: 26774011

[ref19] Edgar R. C. (2013). UPARSE: highly accurate OTU sequences from microbial amplicon reads. Nat. Methods 10, 996–998. doi: 10.1038/nmeth.2604, PMID: 23955772

[ref20] Edgar R. C. Haas B. J. Clemente J. C. Quince C. Knight R. (2011). UCHIME improves sensitivity and speed of chimera detection. Bioinformatics 27, 2194–2200. doi: 10.1093/bioinformatics/btr381, PMID: 21700674 PMC3150044

[ref21] Fijan S. (2014). Microorganisms with claimed probiotic properties: an overview of recent literature. Int. J. Env. Res. Pub. He. 11, 4745–4767. doi: 10.3390/ijerph110504745, PMID: 24859749 PMC4053917

[ref22] Ge X. Wang C. Chen H. Liu T. Chen L. Huang Y.. (2020). Luteolin cooperated with metformin hydrochloride alleviates lipid metabolism disorders and optimizes intestinal flora compositions of high-fat diet mice. Food Funct. 11, 10033–10046. doi: 10.1039/D0FO01840F, PMID: 33135040

[ref23] Guo W. Guo X. J. Xu L. N. Shao L. W. Zhu B. C. Liu H.. (2022). Effect of whole-plant corn silage treated with lignocellulose-degrading bacteria on growth performance, rumen fermentation, and rumen microflora in sheep. Animal 16:100576. doi: 10.1016/j.animal.2022.100576, PMID: 35777297

[ref24] Hayat K. Zhou Y. Menhas S. Bundschuh J. Hayat S. Ullah A.. (2020). *Pennisetum giganteum*: an emerging salt accumulating/tolerant non-conventional crop for sustainable saline agriculture and simultaneous phytoremediation. Environ. Pollut. 265:114876. doi: 10.1016/j.envpol.2020.114876, PMID: 32512425

[ref25] He X. Wang C. Zhu Y. Jiang X. Qiu Y. Yin F.. (2022). Spirulina compounds show hypoglycemic activity and intestinal flora regulation in type 2 diabetes mellitus mice. Algal Res. 66:102791. doi: 10.1016/j.algal.2022.102791

[ref26] Huang Z. Guo W. Zhou W. Li L. Xu J. Hong J.. (2019). Microbial communities and volatile metabolites in different traditional fermentation starters used for Hong Qu glutinous rice wine. Food Res. Int. 121, 593–603. doi: 10.1016/j.foodres.2018.12.024, PMID: 31108786

[ref27] Ingraham R. H. Gillette D. D. Wagner W. D. (1974). Relationship of temperature and humidity to conception rate of Holstein cows in subtropical climate. J. Dairy Sci. 57, 476–481. doi: 10.3168/jds.S0022-0302(74)84917-9, PMID: 4835389

[ref28] Jaber L. S. Duvaux-Ponter C. Hamadeh S. K. Giger-Reverdin S. (2019). Mild heat stress and short water restriction treatment in lactating alpine and Saanen goats. Small Ruminant Res. 175, 46–51. doi: 10.1016/j.smallrumres.2019.04.004

[ref29] Khan A. N. Yasmin H. Ghazanfar S. Hassan M. N. Keyani R. Khan I.. (2021). Antagonistic, anti-oxidant, anti-inflammatory and anti-diabetic probiotic potential of *Lactobacillus agilis* isolated from the rhizosphere of the medicinal plants. Saudi J. Biol. Sci. 28, 6069–6076. doi: 10.1016/j.sjbs.2021.08.029, PMID: 34764740 PMC8568817

[ref30] Kim S. W. Ha Y. J. Bang K. H. Lee S. Bang W. Y. (2020). Potential of Bacteriocins from lactobacillus Taiwanensis for producing bacterial ghosts as a next generation vaccine. Toxins 12:432. doi: 10.3390/toxins12070432, PMID: 32630253 PMC7404994

[ref31] Landry A. P. Cheng Z. Ding H. (2015). Reduction of mitochondrial protein mitoNEET [2Fe–2S] clusters by human glutathione reductase. Free Radical Bio. Med. 81, 119–127. doi: 10.1016/j.freeradbiomed.2015.01.017, PMID: 25645953 PMC4365936

[ref32] Li L. Esser N. M. Ogden R. K. Coblentz W. K. Akins M. S. (2019). Comparison of feeding diets diluted with sorghum-sudangrass silage or low-quality grass on nutrient intake and digestibility and growth performance of Holstein dairy heifers. J. Dairy Sci. 102, 9932–9942. doi: 10.3168/jds.2018-16168, PMID: 31521350

[ref33] Li A. Yang Y. Zhang Y. Lv S. Jin T. Li K.. (2021). Microbiome analysis reveals the alterations in gut microbiota in different intestinal segments of Yimeng black goats. Microb. Pathog. 155:104900. doi: 10.1016/j.micpath.2021.104900, PMID: 33894292

[ref34] Li F. Zhang B. Zhang Y. Hao L. Guo X. (2022). Probiotic effect of feruloyl esterase-producing *lactobacillus plantarum* inoculated alfalfa silage on digestion, antioxidant, and immunity status of lactating dairy goats. Anim. Nutr 11, 38–47. doi: 10.1016/j.aninu.2022.06.010, PMID: 36091259 PMC9404276

[ref35] Li Y. S. Zhou Y. Zhao X. D. Gao F. C. Chen T. D. Jiang J. Q.. (2020). Application of *Pennisetum giganteum* in livestock breeding. Feed Res. 43, 146–148. doi: 10.13557/j.cnki.issn1002-2813.2020.07.037

[ref36] Liu T. Zhou J. Li W. Rong X. Gao Y. Zhao L.. (2020). Effects of sporoderm-broken spores of *Ganoderma lucidum* on growth performance, antioxidant function and immune response of broilers. Anim. Nutr. 6, 39–46. doi: 10.1016/j.aninu.2019.11.005, PMID: 32211527 PMC7082644

[ref37] Ma P. Li Y. Wu J. Mu Q. Li Y. Shu J.. (2020). Effect of differnt silage on production performance and economic benefit of beef. Guizhou Agric. Sci. 48, 87–89. doi: 10.3969/j.issn.1001-3601.2020.03.017

[ref38] Ma T. Suzuki Y. Guan L. L. (2018). Dissect the mode of action of probiotics in affecting host-microbial interactions and immunity in food producing animals. Vet. Immunol. Immunopathol. 205, 35–48. doi: 10.1016/j.vetimm.2018.10.004, PMID: 30459000

[ref39] Metzler B. Bauer E. Mosenthin R. (2005). Microflora management in the gastrointestinal tract of piglets. Asian Austr. J. Anim. 18, 1353–1362. doi: 10.5713/ajas.2005.1353

[ref40] Michels H. (1974). Improvement of livestock production in warm climates: R.E. McDowell. Freeman, San Francisco, Calif., 1972, 711pp., 132 illustrations and 99 tables, £8.40. Livest. Prod. Sci. 1, 225–226. doi: 10.1016/0301-6226(74)90063-3

[ref41] Mu C. T. Ding N. Hao X. Y. Zhao Y. B. Wang P. J. Zhao J. X.. (2019). Effects of different proportion of buckwheat straw and corn straw on performance, rumen fermentation and rumen microbiota composition of fattening lambs. Small Ruminant Res. 181, 21–28. doi: 10.1016/j.smallrumres.2019.09.006

[ref42] Muklada H. Voet H. Deutch T. Zachut M. Kra G. Blum S. E.. (2020). The effect of willow fodder feeding on immune cell populations in the blood and milk of late-lactating dairy goats. Animal 14, 2511–2522. doi: 10.1017/S1751731120001494, PMID: 32638681 PMC7645313

[ref43] National Research Council (2007). Nutrient Requirements of Small Ruminants: Sheep, Goats, Cervids, and New World Camelids. Washington, DC: The National Academies Press.

[ref44] Peng L. Yang Y. F. Hou Y. M. Nu G. D. (2014). The bios*afety asse*ssment of introduced *Pennisetum* sp. in Fujian Province, China. Fujian J. Agr. Sci. 29, 1132–1137. doi: 10.19303/j.issn.1008-0384.2014.11.018

[ref45] Qiu Y. Lei Y. Zhao H. Liu Q. Liu B. (2022). Effects of different microflora of fermented feed of JUNCAO on nutritional quality and antioxidant activity *in vitro*. J. Fujian Agric. Forestry Univ. (Natl. Sci. Edn.) 51, 690–696. doi: 10.13323/j.cnki.j.fafu(nat.sci.).2022.05.016

[ref46] Rashamol V. P. Sejian V. Bagath M. Krishnan G. Bhatta R. (2018). Physiological adaptability of livestock to heat stress: an updated review. J. Anim. Behav. Biometeorol. 6, 62–71. doi: 10.31893/2318-1265jabb.v6n3p62-71

[ref47] Sun T. Miao H. Zhang C. Wang Y. Liu S. Jiao P.. (2022). Effect of dietary *Bacillus coagulans* on the performance and intestinal microbiota of weaned piglets. Animal 16:100561. doi: 10.1016/j.animal.2022.100561, PMID: 35716416

[ref48] Tymensen L. D. Beauchemin K. A. Mcallister T. A. (2012). Structures of free-living and protozoa-associated methanogen communities in the bovine rumen differ according to comparative analysis of 16S rRNA and *mcrA* genes. Microbiology 158, 1808–1817. doi: 10.1099/mic.0.057984-0 22539164

[ref49] Wang J. Yang B. Y. Zhang S. J. Amar A. Chaudhry A. S. Cheng L.. (2021). Using mixed silages of sweet sorghum and alfalfa in total mixed rations to improve growth performance, nutrient digestibility, carcass traits and meat quality of sheep. Animal 15:100246. doi: 10.1016/j.animal.2021.100246, PMID: 34058596

[ref50] Yi J. Dong B. Jin J. Dai X. (2014). Effect of increasing total solids contents on anaerobic digestion of food waste under mesophilic conditions: performance and microbial characteristics analysis. PLoS One 9:e102548. doi: 10.1371/journal.pone.0102548, PMID: 25051352 PMC4106828

[ref51] Zeineldin M. Aldridge B. Lowe J. (2018). Dysbiosis of the fecal microbiota in feedlot cattle with hemorrhagic diarrhea. Microb. Pathog. 115, 123–130. doi: 10.1016/j.micpath.2017.12.059, PMID: 29275129

[ref52] Zeng Y. Hu X. Yu Z. Wang F. Yu F. (2021). Immune enhancement and antioxidant effects of low molecular-weight peptides derived from *Nibea japonica* muscles on immune-deficient mice induced by cyclophosphamide. Proc. Biochem. 102, 42–50. doi: 10.1016/j.procbio.2020.11.016

[ref53] Zheng H. J. Guo J. Wang Q. Wang L. Wang Y. Zhang F.. (2021). Probiotics, prebiotics, and synbiotics for the improvement of metabolic profiles in patients with chronic kidney disease: a systematic review and meta-analysis of randomized controlled trials. Crit. Rev. Food Sci. 61, 577–598. doi: 10.1080/10408398.2020.1740645, PMID: 32329633

[ref54] Zheng J. Wittouck S. Salvetti E. Franz C. M. A. P. Harris H. M. B. Mattarelli P.. (2020). A taxonomic note on the genus *lactobacillus*: description of 23 novel genera, emended description of the genus *Lactobacillus* Beijerinck 1901, and union of *Lactobacillaceae* and *Leuconostocaceae*. Int. J. Syst. Evol. Micr. 70, 2782–2858. doi: 10.1099/ijsem.0.004107, PMID: 32293557

