# Supplementary material for: Effects of fermented feed of Pennisetum giganteum on growth performance, oxidative stress, immunity and gastrointestinal microflora of Boer goats under thermal stress
Source: Front Microbiol. 2023 Jan 12;13:1030262. doi: 10.3389/fmicb.2022.1030262 (PMC9879058; doi:10.3389/fmicb.2022.1030262)
Supplement: Supplementary file 1 [file Table_1.DOCX]

Supplementary Material

**Effects of fermented feed of** ***Pennisetum giganteum* on growth performance, oxidative stress, immunity and gastrointestinal microflora of Boer goats under thermal stress**

**Yuyang Qiu^1^, Hui Zhao^1^, Xiaoyu He^1^, Furong Zhu^1^, Fengli Zhang^1^, Bin Liu^1, 2*^, Qinghua Liu^1, 3*^**

*** Correspondence:** Dr. Bin Liu and Dr. Qinghua Liu, email: [liubin618@hotmail.com](mailto:liubin618@hotmail.com); [83793089@163.com](mailto:83793089@163.com)

**1. Methods for the determination of conventional nutrient composition**

- 1. pH: 10 g of fermented feed was extracted in 90mL distilled water for 0.5 h and then the supernatant was taken for determination by pH meter.
  2. Dry matter (DM): The sample DM value was determined after drying the sample to constant weight in an oven at 105 ± 2°C. Expressed as X /%.
  3. Crude protein (CP): The sample (m) of about 1.000 g was weighed and added into the boiling tube, and then 0.4 g CuSO_4_·5H_2_O, 6.0 g K_2_SO_4_, 10 mL H_2_SO_4_ (Concentration 98.3%), 15 mL H_2_O_2_ (Concentration 30%) were added successively. The samples were digested in an antiboiling apparatus (180 °C for 30 min, 210 °C for 30 min, 360 °C for 30 min, and 400 °C for 70 min), and then cooled to room temperature. Then the Automatic kieldahl apparatus (Kjeltec^TM^ 8400) was used for detection, expressed as X /%.
  4. Crude fiber (CF): The sample (m) of about 0.2000 g was weighed and placed in a glass crucible, which was fixed on crude fiber tester (SLQ-6). 1.25% sulfuric acid solution was added to the glass crucible, which was added to the calibration line, and then a few drops of n-octanol were dropped. Turn on the heater, keep it slightly boiling for 30 min, then filter it, and wash it to neutral with slightly boiling distilled water within 10 min. Then add the NaOH solution (Concentration 1.25%) with a slightly boiling, keep it slightly boiling for 30 min, pump and filter, and wash it with slightly boiling distilled water to neutral within 10 min. Then the samples were washed twice with 15 mL ethanol and then twice with 15 mL petroleum ether. Then the glass crucible was taken out and put into oven, 130 ± 2℃ for 2h. After cooling the constant weight, and the cooling constant weight was recorded as M1. Finally, the glass crucible was placed in a Muffle furnace at 550 ± 20℃ and burned for 30 min. The cooling weight was recorded as M2.

Crude fiber (%) = (M1-M2)/m/ dry matter content ×100%

m: Sample weight (g)

M1: Crucible and sample residue weight after drying at 130℃ (g)

M2: Crucible and sample residue weight after burning at 550℃ (g)

- 1. Ether extract (EE): The sample (m) of about 1.0000 g was weighed, put into the filter paper package and placed in the weighing bottle, and each sample was repeated twice. Put it in the oven at 105 ± 2℃ for 3 ~ 6 h, then take out the measuring bottle and put it in the dryer. Cool it for 1 h and weigh it, which is recorded as W1 (Empty filter paper package + measuring bottle + sample). Besides, the petroleum ether (boiling range 30 ~ 60℃) is poured into the 2/3 height of the siphon, the filter paper package is put into the extraction tube of the Soxhlet extracter with long-handled tweezers and soaked overnight. The condensed water is opened the next day, and heated on the water bath pot at about 50℃. Until the color of the petroleum ether in the extraction tube becomes yellow and colorless, the water bath will stop heating, and this process will take about 10h. Then the filter paper package is taken out and put into the weighing bottle. The weighing bottle is placed in a ventilated place for about 30 min and then put into the oven. The oven door was left open at about 60℃ for 30 min to make all the residual petroleum ether volatilized. After that, the temperature was raised to 105 ± 2℃ for 2 h. The cooling weight was recorded as W2 (Empty filter paper package + weighing bottle + residual sample).

Crude fat content (%) = (W1-W2)/ m/ dry matter content ×100%

m: Sample weight (g)

W1: Empty filter paper package + weighing bottle + sample weight (g)

W2: Empty filter paper package + weighing bottle + weight of residual sample (g)

- 1. Crude ash (CA): The crucible was first put into Muffle furnace and burned at 550 ± 20℃ for 40 min ~ 1 h. After taking it out, the crucible was cooled at room temperature for 1 min and then put into desiccator for 30 min. The crucible weight (W0) was recorded. The crucible with constant weight was used to weigh the sample (m) 2.0000 g, and two replicates of each sample were taken and put into the Muffle furnace. At the beginning of drying, the Muffle furnace door was partially opened, and the temperature of the Muffle furnace was adjusted to about 300℃. After the sample carbonization was completed, the Muffle furnace door was closed. The temperature was set at 550 ± 20℃ for ash, burned for 3 h, and then taken out and cooled in the outside air for about 1 min. Then, it was put into the dryer for cooling and weighing, which was recorded as the weight of crucible + ash (W1).

Crude ash (%) = (W1-W0)/ m/ dry matter content × 100%

m: Sample weight (g)

W0: Constant weight of crucible (g)

W1 Weight of crucible + Ash (g)
